# Herpes Zoster Vaccine Awareness and Uptake Among Older Adults in Saudi Arabia: Insights for Public Health, Preventive Medicine, and Primary Care Practice

**DOI:** 10.1002/jgf2.70115

**Published:** 2026-04-01

**Authors:** Adam F. Aldhawyan, Mohammed A. BuSaad, Hasan M. Alswiket, Abdullah M. Alradhi, Ryad F. Alamry, Fadel A. Almulla, Amjad Z. Boarish, Reema J. Alghamdi, Abdullatif K. Althunyan, Amani M. AlQarni

**Affiliations:** ^1^ Department of Family and Community Medicine, College of Medicine Imam Abdulrahman Bin Faisal University Dammam Saudi Arabia; ^2^ College of Medicine Imam Abdulrahman Bin Faisal University Dammam Saudi Arabia

**Keywords:** attitudes, awareness, herpes zoster vaccine, knowledge, older adults, practices, prevention, Saudi Arabia, shingles, vaccination uptake

## Abstract

**Background/Objectives:**

Complications from Herpes Zoster (HZ) are common among older adults and immunosuppressed populations. The HZ vaccine is a clinically and financially superior alternative to antiviral medications. However, a knowledge gap exists within the population regarding HZ and the vaccine. This quantitative cross‐sectional study assessed the knowledge, practices, and attitudes of people aged > 50 years towards HZ vaccination in the Eastern Province of Saudi Arabia.

**Methods:**

Data were collected via an online self‐administered Arabic questionnaire. It captured sociodemographic and health‐related parameters, knowledge of HZ and its vaccine, and attitudes towards vaccination. A total of 431 responses were collected and a multivariate linear regression was applied to test the association between study variables and participants' awareness about the HZ virus and vaccine.

**Results:**

The participants comprised 53.4% men and 46.6% women with a median age of 54 years (interquartile range 7); 86.5% and 61% of participants were aware of HZ and the vaccine, respectively. The multivariate linear regression model demonstrated that female sex, older age, health insurance, and pox‐history, were significantly associated with greater knowledge of HZ (*p* < 0.05); 85.4% of participants were not vaccinated, citing vaccine side effects (30.2%), low perceived risk (20.9%), and lack of awareness (20.0%). 65.2% and 84.9% of participants expressed interest in learning more about HZ and its prevention, respectively. 69.4% were willing to receive the vaccine if recommended by a healthcare provider.

**Conclusions:**

Despite relatively high awareness, vaccine uptake remained low. These findings highlight the need to explore additional barriers influencing vaccination uptake.

**Abbreviations**
HZ: herpes zoster
IQR: interquartile range
VZV: varicella zoster virus

## 1 Introduction

The varicella zoster virus (VZV) is one of the most contagious diseases. The virus is transmitted via respiratory droplets or direct contact with vesicular varicella lesions causing a primary infection known as chickenpox; which subsequently becomes dormant in nerve tissue and may reactivate later in life. Upon reactivation, VZV spreads along the sensory nerve, manifesting as inflammation and vesicular eruptions in the corresponding dermatome resulting in herpes zoster (HZ); often accompanied by tingling, burning, or aching pain [1]. Several studies highlighted that patients diagnosed with diabetes and those who are immunocompromised face a higher risk of developing HZ as a result of declined cell‐mediated immunity; thus increasing the risk of HZ reactivation [2, 3]. If not adequately treated, HZ may result in various complications such as post‐herpetic neuralgia, which is the most commonly reported, along with zoster associated encephalitis, meningitis, and ophthalmicus [4, 5]. Moreover, HZ infection could be fatal for those who are immunocompromised and the elder population [6].

A systematic review of studies from 2002 to 2018 showed that cumulative incidence HZ has been estimated to be 2.9–19.5 cases per 1000 population worldwide in those aged ≥ 50, with a higher prevalence among females [7] Unfortunately, data on the incidence of HZ in Saudi Arabia and the Middle East in general remain limited. However, a retrospective study conducted at King Faisal Specialist Hospital and Research Center in Riyadh between January 2014 and August 2021 reported a mean incidence rate of 1.4 per 1000 population [2].

An observational cross‐sectional study was conducted to assess the knowledge, attitudes, and practices of the Saudi population regarding HZ and its vaccination [8]. The study findings indicated that only one third of those claiming to have knowledge regarding the HZ vaccine scored 80% or higher when questioned about their knowledge. In addition, only 54.4% of the respondents expressed willingness to receive the vaccine [8]. Similarly, a cross‐sectional study in the United Arab Emirates found that only 1.9% of participants who reported being aware of the HZ vaccine had a high level of knowledge about it, although they generally had positive attitudes towards vaccination [4]. Another cross‐sectional study conducted in Hong Kong involving 408 participants highlighted gaps in knowledge among the participants regarding HZ. Only 29.6% of those participants were aware of the relationship between chickenpox and HZ, and most lacked an understanding of the nature of the disease and its effects. Additionally, 35% of those participants expressed concerns about contracting the disease, whereas only 17% considered vaccination against HZ [9].

The Centers for Disease Control and Prevention recommends that individuals 50 years and older receive two doses of the Shingrix vaccine (GlaxoSmithKline, London, UK), an HZ vaccine [10]. This vaccine has been licensed in Saudi Arabia and is readily available at all primary care centers and is actively recommended by the Saudi Ministry of Heath for adults aged ≥ 50 years of age [11, 12]. However, a recent study conducted in the Western Region of Saudi Arabia revealed a discrepancy. Although Saudi individuals display good knowledge and positive attitudes towards HZ and its vaccination, their uptake is lacking [12].

Numerous studies have been conducted across various regions of Saudi Arabia to examine the knowledge, awareness, and attitudes of the general population and healthcare practitioners regarding HZ and its vaccine. A recently published study from the Eastern Province of Saudi Arabia focused on these objectives using descriptive methodology [13]. However, to date, no studies have focused on identifying factors associated with awareness of HZ and its vaccine in the Eastern Province using multivariable analysis.

Therefore, the primary objective of this study was to assess the knowledge, practices, and attitudes, and their determinants among the general population aged 50 years and older towards HZ vaccination in the Eastern Province, Saudi Arabia.

## 2 Materials and Methods

### 2.1 Study Design and Population

A questionnaire‐based cross‐sectional approach was employed to evaluate the knowledge, attitudes, and practices regarding HZ and its vaccination. The study population included individuals aged ≥ 50 and residing in the Eastern Province of Saudi Arabia in 2023. Individuals younger than 50 years and those not residing in the area were excluded.

Considering an estimated population of 660,000 adults aged ≥ 50 years old, the minimum sample size was calculated to be 384 using the Epi Info tool with a 50% response rate and margin of error and confidence interval of 5% and 95%, respectively. Finally, a total of 431 valid responses were collected and included in the final analysis.

### 2.2 Study Tool

Data were collected using a validated questionnaire from a previously conducted study in the Western Region of Saudi Arabia [12]. This questionnaire consists of 32 closed‐ended questions organized into four sections: sociodemographic data (10 questions), knowledge of HZ and the HZ vaccine (14 questions), and attitudes towards the vaccine (8 questions). The questions include true/false, Likert scale, and multiple‐choice formats. The scoring system implemented is consistent with the scoring system of the original study from which the questionnaire was obtained [12] where a knowledge score for participants' knowledge regarding herpes zoster was calculated based on responses to five questions. Two questions allowed multiple correct responses (six correct selections in total), whereas three questions had one correct answer each. Each correct response was assigned a score of one, while incorrect responses were assigned a score of zero. The total herpes zoster knowledge score therefore ranged from 0 to 9, with higher scores indicating greater knowledge. Similarly, the herpes zoster vaccine knowledge score was calculated based on five questions, with each correct response assigned a score of one and incorrect responses assigned a score of zero, resulting in a possible score ranging from 0 to 5. Because each correct response contributed one point, a one‐point difference in the score represents one additional correct knowledge response [12].

### 2.3 Data Collection

The questionnaire was disseminated via WhatsApp and other social media platforms targeting the intended demographics. Data were collected through an online survey designed using the QuestionPro survey platform. Participation was voluntary, and potential respondents were provided with a clear explanation of the study objectives prior to participation. Participants were assured of the confidentiality of their responses and informed of their right to withdraw from the study at any time without penalty.

Although the survey platform allowed the possibility of multiple submissions, QuestionPro provides functionality for identifying duplicate responses based on Internet Protocol (IP) addresses. During the data cleaning stage, all responses were screened for duplicate IP addresses to identify potential repeat submissions. No duplicate entries were detected; therefore, no responses were excluded from the final dataset.

Sociodemographic and health‐related data were collected including age, gender, region, education level, marital and employment status, presence of chronic illnesses, and prior history of HZ infection. Knowledge‐related items evaluated awareness of the disease, symptoms, transmission, and vaccine eligibility. Attitude questions assessed perceived disease severity, perceived efficacy of vaccination, and trust in healthcare recommendations. Practice items measured prior vaccination status, intent to vaccinate, and sources of vaccine‐related information.

### 2.4 Data Analyses

For descriptive statistics, means and medians were used for continuous variables according to their normality of distribution, whereas frequencies and percentages were used to characterize categorical variables. Chi‐square tests were used to assess significant differences in categorical variables. For continuous variables that did not follow a normal distribution, the Mann–Whitney *U* test was employed for comparisons between two groups and the Kruskal–Wallis test was applied to assess significant differences across multiple groups or strata. Spearman correlation was used to evaluate the relationships between continuous non‐normally distributed variables, and multivariate linear regression was conducted to examine associations between relevant study variables and participant awareness of HZ and its vaccine, while adjusting for other relevant variables. Data analyses were performed using SPSS version 24 (IBM Corp., Armonk, NY, USA) with the significance level set at *p* < 0.05.

### 2.5 Ethical Considerations

Ethical approval for the study was obtained from the Institutional Review Board (IRB) of Imam Abdulrahman Bin Faisal University (IRB number IRB‐UGS‐2023‐01‐361).

## 3 Results

### 3.1 Sociodemographic Characteristics

Table 1 lists the participant sociodemographic and health‐related data. This study included 431 participants: 230 (53.4%) men and 201 (46.6%) women. The median age of the participants was 54 (interquartile range [IQR] 7) years. Most of the sample comprised Saudi participants (92.8%). Of the participants, 161 (37.4%) reported having had chickenpox, 167 (38.7%) did not ever have it, and 103 (38.7%) were unable to recall. Regarding HZ awareness, 373 (86.5%) participants were aware of HZ, 263 (61.0%) were aware of the HZ vaccine, and the remaining participants were unaware of the vaccine.

**TABLE 1:** Participant sociodemographic and health‐related data (*N* = 431).

Characteristic	Category	*N* (%)
Sex	Men	230 (53.4%)
	Women	201 (46.6%)
Age in years	Median (interquartile range)	54 (7)
Nationality	Saudi	400 (92.8%)
	Non‐Saudi	31 (7.2%)
Occupation	Unemployed	107 (24.8%)
	Employed	163 (37.8%)
	Retired	161 (37.4%)
Residency	City	406 (94.2%)
	Village	25 (5.8%)
Health insurance	Yes	167 (38.7%)
	No	264 (61.3%)
Monthly income	Less than 5000 SAR	90 (20.9%)
	5000–9999 SAR	103 (23.9%)
	10,000–19,999 SAR	145 (33.6%)
	20,000 or more	93 (21.6%)
Education level	Intermediate school or less	44 (10.2%)
	High school	96 (22.3%)
	Diploma	49 (11.4%)
	Bachelor's degree	190 (44.1%)
	Postgraduate education	52 (12.1%)
Have chronic diseases	Yes	271 (62.9%)
	No	160 (37.1%)
Chronic diseases	Hypertension	120 (27.8%)
	Diabetes Miletus	111 (25.8%)
	Hypercholesterolemia	102 (23.7%)
	Chronic obstructive pulmonary disease	4 (0.93%)
	Hypothyroidism	31 (7.2%)
	Gout	18 (4.2%)
	Osteoarthritis	29 (6.7%)
	Rheumatoid arthritis	29 (6.7%)
	Coronary artery disease	3 (0.7%)
	Mental disorders	15 (3.5%)
	Asthma	20 (4.6%)
	None	160 (37.1%)
	Others	31 (7.2%)
Medications	Cortisone	24 (5.6%)
	Antirheumatic medications	17 (3.9%)
	Biological treatment	10 (2.3%)
	Chemotherapy	5 (1.16%)
	Radiotherapy	3 (0.7%)
	None	388 (90.02%)
Have had chicken pox	Yes	161 (37.4%)
	No	167 (38.7%)
	Cannot recall	103 (23.9%)
Have heard about HZ	Yes	373 (86.5%)
	No	58 (13.5%)
Have heard about HZ vaccine	Yes	263 (61.0%)
	No	168 (39.0%)
SAR, Saudi riyal.		

### 3.2 HZ Knowledge and Awareness

Table 2 presents the relationship between HZ awareness and sociodemographic characteristics. Awareness of HZ was significantly associated with specific sociodemographic characteristics. Sex exhibited a significant association with HZ awareness, as 91% of the women and 82.6% of the men were aware of HZ (*p* = 0.010). Educational level was also significantly associated with HZ awareness. Those with a bachelor's degree or higher exhibited better awareness (90.5%) than those with a high school diploma or lower educational level (81.5%) (*p* = 0.031). Conversely, other sociodemographic characteristics did not demonstrate a significant association with HZ awareness.

**TABLE 2:** Associations between sociodemographic data and participant awareness and knowledge scores about HZ, including multivariate regression analysis (*N* = 431).

Parameter	Category	Awareness about HZ (Yes: N (%))	Awareness *p*‐value (a)	Knowledge Score Median (IQR)	Knowledge *p*‐value (b)	Regression beta	95% confidence interval	Regression *p*‐value
Sex	Men	190 (82.6%)	0.010	3 (5)	< 0.001	Reference		
	Women	183 (91%)		4 (4)		0.17	(0.34, 1.61)	0.003
Age in years		Aware: 54.00 (7.00), Unaware: 55.00 (8.00)	0.114	3 (5)	0.079	0.11	(0.01, 0.12)	0.030
Nationality	Saudi	347 (86.8%)	0.590	3 (5)	0.758	Reference		
	Non‐Saudi	26 (83.9%)		3 (6)		−0.01	(−1.21, 1.10)	0.925
Occupation	Unemployed	96 (89.7%)	0.539	4 (4)	0.023	Reference		
	Employed	139 (85.3%)		3 (4)		−0.10	(−1.48, 0.270)	0.176
	Retired	138 (85.7%)		4 (5)		−0.03	(−1.06, 0.64)	0.629
Residency	City	353 (86.9%)	0.360	3 (5)	0.140	Reference		
	Village	20 (80%)		3 (4.5)		−0.07	(−1.96, 0.32)	0.159
Health insurance	Yes	141 (84.4%)	0.307	4 (5)	0.028	0.18	(0.49, 1.72)	< 0.001
	No	232 (87.9%)		3 (4)		Reference		
Monthly income	< 5000 SAR	74 (82.8%)	0.604	3 (5)	0.702			
	5000–9999 SAR	90 (87.4%)		3 (4)				
	10,000–19,999 SAR	127 (87.6%)		3 (5)				
	20,000 or more	82 (88.2%)		4 (5)				
Education level	Intermediate school or less	33 (75%)	0.031	4 (4)	0.006	Reference		
	High school	82 (85.4%)		2.5 (5)		−0.14	(−1.96, 0.06)	0.066
	Diploma	39 (79.6%)		3 (4)		−0.05	(−1.62, 0.79)	0.501
	Bachelor's degree	173 (91.1%)		3 (4)		0.03	(−0.83, 1.18)	0.732
	Postgraduate education	46 (88.5%)		5 (6.75)		0.10	(−0.35, 2.17)	0.158
Have had chicken pox	Yes	142 (88.2%)	0.268	5 (4)	< 0.001	0.12	(0.15, 1.31)	0.014
	No	139 (83.2%)		3 (4)		Reference		
	Cannot recall	92 (89.3%)		3 (5)		0.01	(−0.75, 0.63)	0.866

The calculated HZ knowledge scores had a median (IQR) of 3 (5) (not displayed in the tables) and displayed significant associations with multiple sociodemographic characteristics (Table 2). Similar to HZ awareness, women had a higher median (IQR) HZ knowledge score of 4 (4) compared to the 3 (5) for men (*p* < 0.001). Interestingly, employed participants had a lower median (IQR) knowledge score of 3 (4) than the 4 (5) for those unemployed and retired (*p* = 0.023). Participants with health insurance had a higher knowledge score of 4 (5) than the 3 (4) for those without health insurance (*p* = 0.028). Education levels exhibited significant associations with higher median (IQR) knowledge scores in the following order: postgraduate education 5 (6.75), intermediate school or lower with 4 (4), high school diploma and bachelor's degree 3 (4), and high school with 2.5 (5) (*p* = 0.006). Chickenpox history was an indicator for a higher median (IQR) knowledge score (*p* < 0.001). The remaining sociodemographic characteristics presented in Table 2 did not have a significant association with HZ knowledge scores.

The multivariate linear regression analysis identified several predictors positively associated with high HZ knowledge scores (Table 2). These predictors included women (*β* = 0.17, *p* = 0.003), age (*β* = 0.11, *p* = 0.030), health insurance (*β* = 0.18, *p* < 0.001), and chickenpox history (*β* = 0.12, *p* = 0.014). On the other hand, other variables were not found to be significantly associated with the knowledge scores.

**FIGURE 1:**
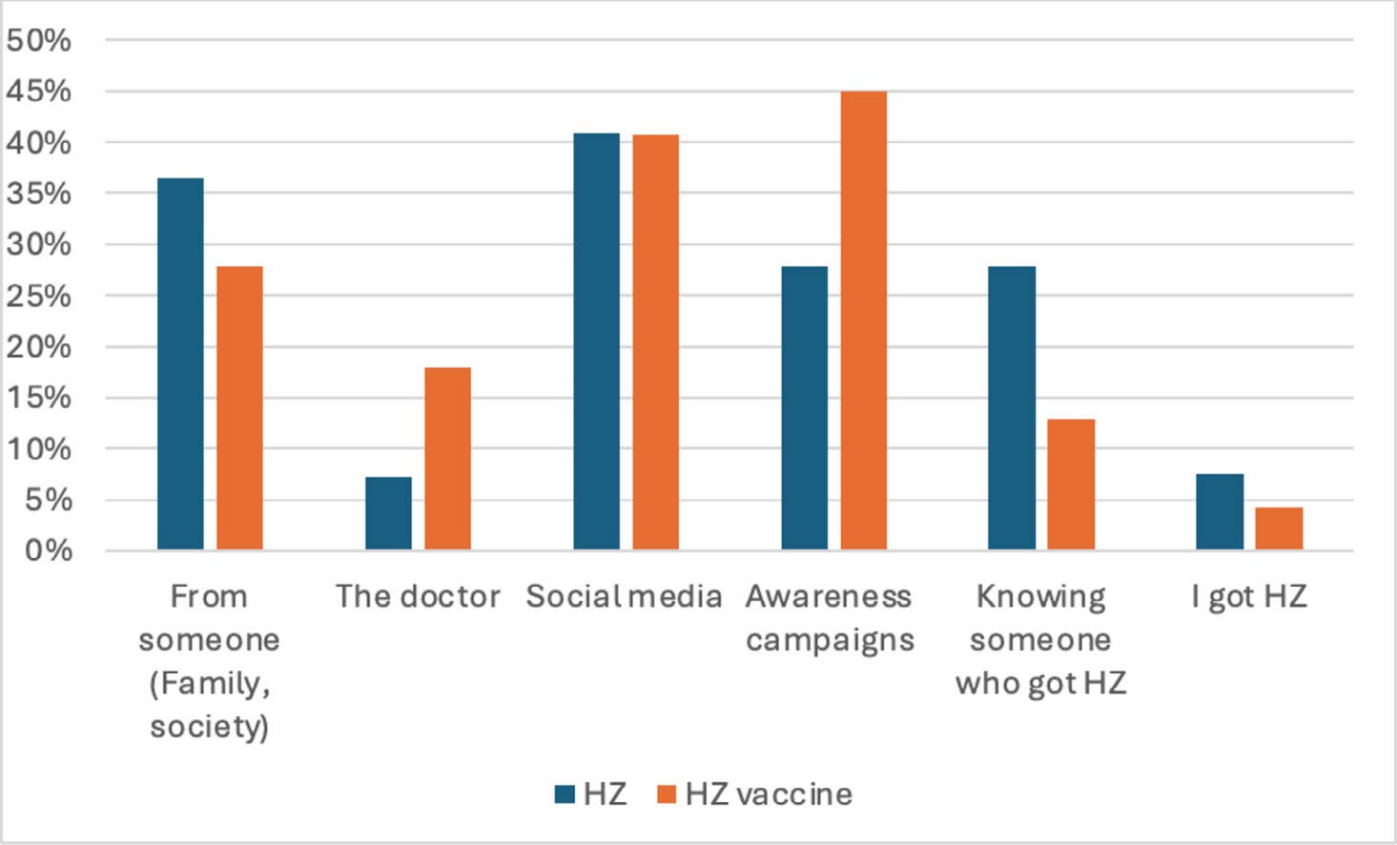
Sources of knowledge about HZ and HZ vaccine for participants aware of it and the vaccine.

Figure 1 illustrates the different sources of knowledge regarding HZ and the HZ vaccine for participants who were aware of the disease and its vaccine. Social media (40.8%), family and society (36.5%), and personal connections with individuals who had experienced HZ (27.9%) emerged as the most prevalent sources of awareness. However, knowledge about the vaccine seemed to be obtained from medical sources (44.9% campaigns and 17.9% doctors), relatives, and society (27.8%).

### 3.3 HZ Vaccine Knowledge and Awareness

None of the sociodemographic characteristics exhibited significance regarding awareness of the HZ vaccine. Only a higher level of income was associated with a higher knowledge score (*p* = 0.005). The multivariate linear regression analysis conducted to identify predictors for higher HZ vaccine knowledge scores indicated no significant predictors and the overall model was not significant. Further details are in Tables A1 and A2.

### 3.4 Attitudes and Practice Towards HZ Vaccine

Table 3 lists the practices and attitudes towards the HZ vaccine. The results revealed with 85.4% of the participants did not receive the vaccine, 6.3% received a single dose, and only 8.4% completed the two recommended doses. The three most common reasons for not receiving the vaccine were concerns about vaccine side effects (30.2%), a belief of being at low risk of developing HZ (20.9%), and lack of awareness about the vaccine (20.0%).

**TABLE 3:** Practice and attitudes towards HZ vaccine (*N* = 431).

Characteristic	Category	*N* (%)
Ever received HZ vaccine	Yes (Single dose)	27 (6.3%)
	Yes (Two doses)	36 (8.4%)
	No	368 (85.4%)
Reasons for not receiving the vaccine	I am not at risk of getting HZ because I am in good health	90 (20.9%)
	I do not believe in vaccines	48 (11.1%)
	I prefer taking medications when getting HZ	62 (14.4%)
	I am worried about the side effects of the vaccine	130 (30.2%)
	I did not know about the vaccine	86 (20.0%)
	I think it is waste of money	5 (1.2%)
	I think the vaccine is very expensive	6 (1.4%)
	It is not covered by health insurance	10 (2.3%)
	None (I received/will receive the vaccine)	134 (31.1%)
	Others	19 (4.4%)

**FIGURE 2:**
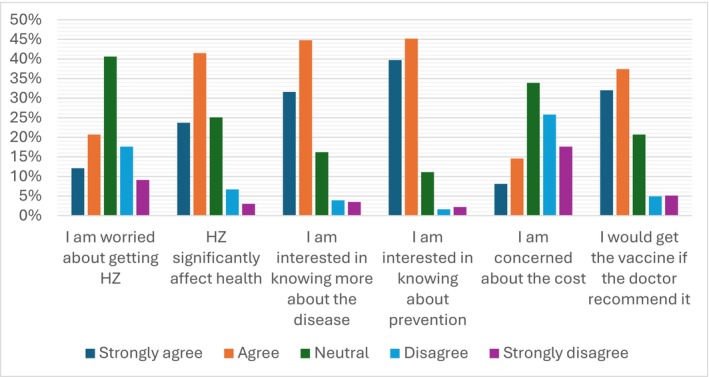
Participant attitudes towards HZ and its vaccine (*N* = 431).

However, it is worth noting that the participants had a positive attitude towards the HZ vaccine, as shown in Figure 2. The majority of participants agreed or strongly agreed that they were interested in learning more about HZ (65.2%) and its prevention (84.9%). Furthermore, 69.4% of the participants expressed their willingness to receive the vaccine if recommended by their doctors.

## 4 Discussion

This study revealed a relatively high level of awareness regarding HZ and the HZ vaccine among the participants; significantly higher levels were observed among those who were women, older, with health insurance, and those with a chickenpox history. In addition to identifying the levels of knowledge, these results make a valuable contribution to the existing literature as it examines various factors associated with HZ knowledge among the Eastern Province population using a multivariable analysis; providing further insight into the characteristics shaping the public's knowledge and attitudes.

In this study, 86.5% of the participants who are residing in the Eastern Province were aware of HZ. Despite a positive attitude, a large percentage of participants (85.4%) remained unvaccinated. Although awareness levels in this study were only slightly higher than other nationwide studies conducted in Saudi Arabia, including the Western Region and Al‐Ahsa [1, 4, 12, 14], vaccination uptake remained low across the different regions. Moreover, a study that compared all Saudi Arabian regions showed contrasting findings, reporting 68.8% awareness regarding HZ in the Eastern Province [8]. However, that study may not have been reliable for generalization as it only included 48 participants from the Eastern Province [8]. Not all of those studies calculated a knowledge score; nonetheless, our participant knowledge scores median (3) and IQR (5) are close to the calculated knowledge score median for the participants of the Western Region study, which are 4 and IQR (3 to 5); however, the methods for calculating the knowledge score were slightly different between the two studies [12].

When comparing our results to international studies, our results have also showed a higher level for awareness. A study conducted by Yang et al. in the Republic of Korea found that 85.7% of their population had heard of HZ, which is consistent with our findings [15]. Unlike our study, their study did not exclude participants younger than 50 years, which can limit comparisons between the two studies; in particular, their study found a significant association between HZ awareness and younger participants. However, awareness of HZ for our participants was 86.5%, which is considerably higher than that of the United Arab Emirates population of 64.3% [4].

Gaining insights into the factors associated with increased awareness and knowledge of HZ is crucial for effective implementation of awareness‐raising methods. In this study, the factors associated with higher awareness and knowledge included female sex, higher education level, having health insurance, chickenpox history, and being unemployed or retired. A possible explanation for the association between higher awareness and knowledge of HZ among insured individuals with a higher level of education is that having health insurance may provide better access to healthcare resources, including information about diseases such as HZ. Moreover, higher education is often associated with a broader knowledge base and better understanding of health‐related topics [16]. However, some factors are difficult to explain, such as sex, although studies that assessed HZ awareness and knowledge have indicated women are more aware and knowledgeable than men [4, 8, 12, 15]. Additionally, it seems that these results are not specific to HZ because another study that evaluated awareness levels for coronavirus disease among Saudis showed similar higher awareness levels among women [17]. Interestingly, in contrast to our findings, a study conducted in the Western Region found that being employed or retired was associated with a higher level of awareness than being unemployed [12]. Other factors such as sex, education level, and chickenpox history are generally similar among the studies [4, 8, 12, 15].

Our participants again demonstrated a better awareness level than those in other studies [4, 8, 9, 12]. Although the awareness level for vaccination was less than the awareness level for the disease, 61.0% of the participants were aware of the HZ vaccine, whereas the rates were 55.8% for the Western Region residents who were aware [12], 57.2% for the study conducted by AlMuammar et al. [18], and 51.6% for the study conducted by Alleft et al. [8]. When compared to the Emirati and Korean populations, the differences become more significant; only 14.8% of the Emirati population and 43.6% of the Korean population were aware of the HZ vaccine [4, 15]. Knowledge scores were relatively low, with a median of 1 in both our study and a study conducted in the Western Region [12]. The factors that indicated a higher awareness level were similar to those identified for disease awareness in most of the studies [4, 8, 12]. Our study only identified higher monthly income as a potential indicator of higher knowledge scores, although this did not reach significance in the regression model.

Evaluating participant sources of knowledge about HZ and its vaccine was a major objective of our study and other studies because it is important to identify optimal ways of implementing interventions to raise awareness. Unsurprisingly, the internet, exemplified by social media, formed a major source of HZ and HZ vaccine awareness among our participants (40.8%). Similarly, in the studies conducted in Al‐Ahsa [14], Western Region [12], as well as in the study by AlMuammar et al. [18], the internet was a source of knowledge for 30.5%, 24.5%, and 22.6% of the participants, respectively. The internet has become a widespread modality for health information, as indicated by 90.9% of the participants in a recent study conducted in Saudi Arabia [19]. This highlights the importance of ensuring credible information is provided in social media. In addition to the internet, awareness campaigns were reported by the participants as a source of information regarding HZ and its vaccine, accounting for 27.9% of the participants in our study. Adjustments in the contents of awareness campaigns may be needed because the knowledge scores in our study and other studies indicated a limitation in delivering health information in those campaigns. Furthermore, the high percentage of awareness campaigns might explain the reason women were more aware than men because a study conducted in Saudi Arabia found that women were more inclined to attend awareness campaigns [20].

A concerning finding in our study was the very low percentage of doctors as an information source for HZ (7.2%) and the HZ vaccine (17.9%). This finding is not exclusive to our study; another local study conducted in the Western Region of Saudi Arabia had similar results [12]. Time constraints and a high flow of cases might limit physicians' ability to allocate time for health education, underscoring the need to establish dedicated health education and preventive medicine clinics, specifically for HZ education. Additionally, this might indicate less understanding and consideration about HZ as an important clinical condition. This assumption is supported by a study conducted among primary healthcare providers working in Mekkah, Saudi Arabia, which found that only 80.4% of the physicians were aware of HZ, which is even lower than our participant’ awareness levels [21]. Furthermore, a study exploring physicians' knowledge, attitudes, and practices regarding the HZ vaccine showed that only 66% of the physicians considered the HZ vaccination an important clinical priority [22]. This highlights the importance of raising awareness of HZ vaccination not only in the general population, but also in healthcare workers.

Fortunately, our participants' attitudes were satisfactory, with 65.2% interested in learning more about HZ and 84.9% interested in its prevention. This has been observed in almost all studies conducted in this area of research [4, 8, 12, 15, 18]. Additionally, the majority of our participants and those in other studies were willing to receive the vaccine if recommended by their doctors [4, 8, 12, 15, 18]. This highlights the need for efforts to convince the population be vaccinated, increase their knowledge, and correct misconceptions, especially because 89% of the participants of the study in Al‐Ahsa believed in the effectiveness of the HZ vaccine [14].

In our study, only 6.3% of the patients received a single dose, and only 8.4% completed the two recommended doses. This is consistent with all other studies, in that none of those studies exceeded the percentage of vaccinations of our participants [4, 12, 14, 18]. One of the major reasons for avoiding vaccination is concerns about side effects, both in our study and in other studies. Notably, serious side effects of Shingrix, which is the most commonly administered vaccine against HZ, are extremely rare; the side effects experienced are typically mild to moderate pain, redness, and swelling in the arm, myalgia, fever, and shivering that usually resolve within 2–3 days [23]. In addition to concerns about side effects, lack of awareness and belief in being at low risk of developing HZ were major concerns, and all of these barriers can be overcome through appropriate awareness campaigns.

These results may suggest that focusing on raising the public's awareness and access to reliable health information regarding HZ and its vaccine among both the general population and physicians may be beneficial, particularly by using commonly used sources such as social media and awareness campaigns as identified by participants. In particular, awareness campaigns should focus on correcting misconceptions about HZ and the HZ vaccine using plain language to ensure good understanding of the health materials. Ensuring credible information is presented in social media is critically important to avoid further misconceptions.

### 4.1 Strengths and Limitations

Several studies in Saudi Arabia have explored the public's knowledge and attitudes regarding HZ and its vaccine. This study further examines the determinants of HZ knowledge in the Eastern Province by identifying sociodemographic and health‐related factors associated with such knowledge using a multivariable analytical approach. This study has several strengths, including an adequate sample size, a focus on the Eastern Province, and a comprehensive assessment of HZ and its vaccine.

However, certain limitations must be acknowledged. First, participants were predominantly recruited through social media platforms, particularly WhatsApp groups. This recruitment method may have introduced selection bias, as social media users may possess higher health awareness than the general population. Consequently, this may have influenced the prominence of social media as the most common source of knowledge among participants. Second, the reliance on self‐reported history of chickenpox introduces a potential source of recall bias. Employing more objective methods of data collection could have enhanced the robustness of the findings. Lastly, the regression model explained a relatively small proportion of the variation in HZ knowledge, suggesting that additional unmeasured factors may contribute to differences in knowledge within the population.

### 4.2 Conclusion and Public Health Implications

The findings of this study demonstrate that although awareness and attitudes towards HZ and its vaccine were relatively high among participants, vaccine uptake remained low. This discrepancy suggests that awareness alone may not necessarily translate into vaccination behavior and highlights the importance of understanding additional factors that influence vaccination decisions.

More broadly, vaccination behavior is likely influenced by multiple determinants, including individual, social, cultural, and health‐system factors. Consequently, improving knowledge alone may not be sufficient to drive behavioral change, and coordinated strategies addressing these different determinants may be required to enhance vaccine uptake.

From a public health perspective, structural barriers such as inconsistent healthcare provider engagement, fragmented care pathways, and gaps in public awareness have been identified as challenges to adult immunization in Eastern Mediterranean countries, including Saudi Arabia [24]. Addressing these barriers may require integrated strategies that combine public awareness initiatives, strengthened healthcare provider engagement, and improved integration of adult vaccination into routine preventive care. Such efforts align with Saudi Arabia's Vision 2030 strategy, which emphasizes community‐based care and proactive disease prevention. Future studies are warranted to further explore the range of factors influencing vaccination decisions and to better inform targeted interventions.

## Author Contributions

**Adam F. Aldhawyan:** conceptualization, validation, resources, writing – original draft, writing – review and editing, visualization, supervision, project administration, funding acquisition. **Mohammed A. BuSaad:** validation, resources, writing – original draft, writing – review and editing, visualization, supervision, project administration, funding acquisition. **Hasan M. Alswiket:** software, formal analysis, resources, writing – original draft, writing – review and editing, funding acquisition. **Abdullah M. Alradhi:** software, formal analysis, data curation, writing – original draft, writing – review and editing. **Ryad F. Alamry:** methodology, formal analysis, resources, data curation, writing – original draft, writing – review and editing, funding acquisition. **Fadel A. Almulla:** methodology, investigation, resources, data curation, writing – original draft, writing – review and editing, funding acquisition. **Amjad Z. Boarish:** conceptualization, methodology, investigation, resources, data curation, writing – original draft, writing – review and editing, funding acquisition. **Reema J. Alghamdi:** writing – original draft, writing – review and editing. **Abdullatif K. Althunyan:** writing – review and editing, project administration. **Amani M. AlQarni:** resources, writing – review and editing, visualization, funding acquisition. All authors have read and agreed to the published version of the manuscript.

## Funding

The authors have nothing to report.

## Ethics Statement

The study was conducted in accordance with the Declaration of Helsinki, and approved by the Institutional Review Board (IRB) of Imam Abdulrahman Bin Faisal University (IRB number IRB‐UGS‐2023‐01‐361).

## Consent

Informed consent was obtained from all subjects involved in the study.

## Conflicts of Interest

The authors declare no conflicts of interest.

## Data Availability

The data that support the findings of this study are available from the corresponding author upon reasonable request.

## Appendix A

**TABLE A1:** Associations between sociodemographic data and participant awareness and knowledge scores about the HZ vaccine (*N* = 431).

Parameter	Category	Awareness about HZ vaccine		HZ vaccine knowledge score	
		Yes: *N* (%) or Median (IQR)	*p* ^a^	Median (IQR)	*p* ^b^
Sex	Men	138 (60%)	0.642	1 (2)	0.693
	Women	125 (62.2%)		1 (2)	
Age in years	Aware about vaccine	54.00 (6.00)	0.059	Age: 54 (7) Knowledge score: 1 (2)	0.607
	Unaware about vaccine	54.00 (7.00)			
Nationality	Saudi	249 (62.3%)	0.060	1 (2)	0.601
	Non‐Saudi	14 (45.2%)		1 (2)	
Occupation	Unemployed	63 (58.9%)	0.384	1 (2)	0.625
	Employed	95 (58.3%)		1 (2)	
	Retired	105 (65.2%)		1 (2)	
Residency	City	247 (60.8%)	0.753	1 (2)	0.329
	Village	16 (64%)		0 (2.5)	
Health insurance	Yes	98 (58.7%)	0.429	1 (2)	0.588
	No	165 (62.5%)		1 (2)	
Monthly income	Less than 5000 SAR	51 (56.7%)	0.487	0 (2)	0.005
	5000‐9999 SAR	59 (57.3%)		1 (2)	
	10,000–19,999 SAR	94 (64.8%)		1 (2)	
	20,000 or more	59 (63.4%)		2 (3)	
Education level	Intermediate school or less	26 (59.1%)	0.225	0.5 (2)	0.187
	High school	51 (53.1%)		0 (1)	
	Diploma	27 (55.1%)		0 (1)	
	Bachelor's degree	126 (66.3%)		1 (2)	
	Postgraduate education	33 (63.5%)		1 (3)	
Have had chicken pox	Yes	100 (62.1%)	0.722	1 (2)	0.980
	No	98 (58.7%)		1 (2)	
	Cannot recall	65 (63.1%)		1 (2)	

Abbreviations: IQR, interquartile range; SAR, Saudi riyal.

^a^ Chi‐square test, Fisher's exact, or Mann–Whitney *U* test was used to calculate the *p*‐value.

^b^ Mann–Whitney *U* test, Kruskal–Wallis test, Spearman correlation was used to calculate the *p*‐value.

**TABLE A2:** Multivariate linear regression analysis for herpes zoster vaccine knowledge predictors.

Parameter	Category	Herpes zoster knowledge		
		Beta	95% confidence interval	*p*
Sex	Men	Reference		
	Women	−0.03	(−0.49, 0.27)	0.577
Age in years		−0.02	(−0.04, 0.03)	0.683
Nationality	Saudi	Reference		
	Non‐Saudi	−0.01	(−0.75, 0.64)	0.883
Occupation	Unemployed	Reference		
	Employed	−0.03	(−0.62, 0.43)	0.717
	Retired	0.03	(−0.41, 0.62)	0.691
Residency	City	Reference		
	Village	−0.05	(−1.03, 0.34)	0.32
Education level	Intermediate school or less	Reference		
	High school	−0.02	(−0.70, 0.53)	0.783
	Diploma	−0.02	(−0.83, 0.62)	0.776
	Bachelor's degree	0.10	(−0.29, 0.92)	0.302
	Postgraduate education	0.11	(−0.19, 1.32)	0.144
Insurance	No	Reference		
	Yes	0.04	(0.49, 1.72)	0.398
Have had chicken pox	No	Reference		
	Yes	−0.00	(−0.38, 0.37)	0.978
	Cannot recall	0.01	(−0.36, 0.48)	0.780
